# Oral Health of Children from the SOS Children’s Village in Croatia

**DOI:** 10.3390/ijerph18020616

**Published:** 2021-01-13

**Authors:** Zrinka Ivanisevic, Zvonimir Uzarevic, Stjepanka Lesic, Aleksandar Vcev, Marko Matijevic

**Affiliations:** 1 Department of Dental Medicine, Faculty of Dental Medicine and Health, University of Osijek, 31000 Osijek, Croatia; zrinkaivan@gmail.com (Z.I.); stjepankaovic@yahoo.com (S.L.); aleksandar.vcev@fdmz.hr (A.V.); 2 Department of Natural Sciences, Faculty of Education, University of Osijek, 31000 Osijek, Croatia; zuzarevic@foozos.hr

**Keywords:** caries, children, SOS children’s village

## Abstract

The aim of this study was to determine the values of DMFT/DMFS and dft/dfs in the examined groups of children and the assessment of the mothers of the examined groups of children related to the oral health of their children. The research included children from the SOS Children’s Village in Croatia as well as children from biological families from rural and urban areas. The children were examined by the visual–tactile method according to the standardized World Health Organization criteria. dft/DMFT and dfs/DMFS indices were calculated. An analysis of completed questionnaires was made. The children from the SOS Children’s Village demonstrated the lowest mean values of the dft/dfs (2.42/3.31) and DMFT/DMFS (1.61/2.23) indices compared to children from rural and urban areas. The Kruskal–Wallis test showed a significant difference (*p* = 0.01) in SiC index values between the examined children. In the groups of children from the SOS Children’s Village and from the rural area compared to the children from the urban area, oral hygiene was singled out as the most important factor in the analysis of the main components. An equally significant factor for all the respondents is the assessment of oral health and eating habits. The least significant factor for the group of children from the SOS Children’s Village is socio-economic status, which is the most significant for the children from the urban area. The children from the SOS Children’s village have the lowest dft/DMFT, dfs/DMFS, and SiC indices. The most important factor influencing oral health in the group of children from the SOS Children’s Village that stands out is oral hygiene, and the least important is the socio-economic status. The assessment of oral health by the SOS mothers does not differ from the assessment of biological mothers of children from rural and urban areas.

## 1. Introduction

Oral health is an integral part of general health and an important factor in the overall quality of life. In spite of the great efforts made to preserve oral health, not only in Croatia but also worldwide, oral cavity diseases are on the rise [1,2]. The family plays an important role in the development of every child and, therefore, also in the formation of children’s attitudes related to health, since health behavior of parents definitely affects the behavior of children. Responsible behavior related to health implies good information and the habit of practicing proper oral hygiene. A healthy and stimulating family provides health care and childcare, is connected to the social community, and includes children in organizations outside the family, where they learn working habits and acquires life values. It has been proven that the economic power of the family also affects the health and emotional stability of children [3].

Oral health literacy is the ability to understand and properly use the information, instructions, and guidelines related to oral cavity health, and it includes the knowledge and practice of oral-hygiene measures, the recognition of risk factors affecting oral health, the education on different aspects of oral health, the awareness of the link of general and oral health as well as their mutual influence on the quality of life, as well as the construction and maintenance of various traditional and modern communication methods between patients and dentists with the aim of raising oral health literacy [4].

The research in the Netherlands and Australia studies the influence of parents on the development of children’s oral hygiene by knowledge transfer, but also by the control of children’s heath behavior [5,6]. The research conducted in Finland in 2007 with the population of children aged 11 to 12 showed that the factors related to oral health and parents’ behavior have a greater influence on girls, considering the development of initial caries, while fathers’ poor oral hygiene proved significant for caries development in boys [7]. Wigen and Wang noticed that the social environment in which a child develops and grows is related to caries development. Additionally, they showed that the importance of parents’ education and free dental care are an important factor for low caries prevalence in the territory of Norway [8].

By transferring certain knowledge and experiences, and primarily by educating parents, we can influence the appropriate health behavior of children. While there are years of education and gaining competence, profession wise, there are no classes on being an adequate parent. So when it comes to parenting models, it all comes down to intuition, or possibly some knowledge and experience. Interestingly enough, there is no evaluation process that a parent has to go through, even though the parental influence plays the biggest role in children’s lives, in comparison with the elaborated competence evaluation and self-reflection that the experts who deal with families and children have to go through [9].

SOS Children’s Village is a leading organization in the field of alternative childcare. This well-organized community cares for children without parents or parental care regardless of their racial, national, and religious affiliation, providing them with love and security in a family environment. The first SOS Children’s Village was founded by Hermann Gmeiner (1919–1986) in the Austrian town of Imst in 1949. Today, SOS-Kinderdorf International is the umbrella organization that brings together 133 countries with 533 SOS Children’s Villages. SOS Children’s Village operates in the spirit of the United Nations Convention on the Rights of the Child by promoting this right worldwide. The development of a child in a caring family environment, a lasting home, and education for a happy and peaceful childhood is supported by the realization of the following rights: the right to care, education, health (preventive and active health care), and psychosocial support. SOS Children’s Village in the territory of Croatia has been present for twenty-five years. Mothers play a key role in children’s lives in the SOS Children’s Village, providing them with a home and a stable family environment. SOS mothers go through a careful selection process and a long-term training, and they meet all the physical and emotional needs of children. One of the elements of a healthy psychophysical development of children is oral health care, and the specific life conditions in this environment (child–SOS mother) at an early age can influence not only psychophysical growth and development, but also the state of oral health. So far, very little data have been published in the literature on the oral health of children in this population, and there is no data on the oral health of children living in SOS Children’s Villages [10,11]. The aim of this study was to determine the values of DMFT/DMFS and dft/dfs in the examined groups of children and the assessment of the mothers of the examined groups of children related to the oral health of their children.

## 2. Subjects and Methods

### 2.1. Respondents

A total of 218 primary school children from the SOS Children’s Village, 224 primary school children from biological families in rural areas, and 178 primary school children from biological families in urban areas were examined in the period from February 2018 to October 2019. The criteria for the inclusion of respondents were as follows: all primary school children from the SOS Children’s Village and randomly selected children from rural and urban areas. The exclusion criteria for conducting this research were children younger than six and older than 14 and children with craniofacial anomalies (clefts and syndromes). The identity of the respondents was protected by using an identification number instead of the name and surname of each respondent. Prior to the dental examination, all children included in the research had their body weight and height measured in order to calculate their body mass index (BMI). Parents/guardians signed an informed consent. The children were examined in dental offices according to standards of the World Health Organization (WHO) in conditions of controlled hygiene and using appropriate lighting. To conduct this research, a permit was obtained by the Ethics Committee for Research of the Josip Juraj Strossmayer University of Osijek, Faculty of Medicine (class: 602-04/13-08/09, number: 2158-61-07-13-45).

### 2.2. Methods

#### 2.2.1. Visual–Tactile Examination

The clinical approach to the research was the same for all the children, involving a visual–tactile examination of the oral cavity using a probe, a mirror, and an air syringe [12]. The examination was performed by the same examiner with the help of an assistant who recorded oral status data in the pre-prepared forms created according to the 1997 WHO method [13]. Every tooth (or surface) that by sounding and visual inspection showed signs of lesions in pits, fissures, or walls (cavities, undermined enamel, finding of softened walls) was recorded as carious. The evaluation was determined according to WHO criteria, and the information on each tooth was recorded using codes [13]. In this paper, the following indices were calculated: DMFT/dft index (Decayed/decayed, Missing, Filled/filled, Tooth/tooth), DMFS/dfs index (Decayed/decayed, Missing, Filled/filled, Surfaces/surfaces), and SiC index (Significant Caries) index [14]. The calibration of the examiner was done in a way that the researcher examined 30 children of different age two months before and immediately before the study in which the kappa value was 0.95.

#### 2.2.2. Questionnaire

Apart from the clinical examination, an analysis of a customized and culturally adapted questionnaire on oral health knowledge, socioeconomic status, eating habits, and oral hygiene was performed [15]. The questionnaire was completed by mothers/guardians of children in order to determine the existence of specific socioeconomic, eating, and health–hygienic indicators in the population. Immediately before the distribution of the questionnaire, the examiners were taught how to record the data in the questionnaire. The questions in the questionnaire for mothers/guardians were divided into thematic groups. The questionnaire included questions about the socioeconomic status of the family (highest level of education and employment status, the number of persons, and household income) and questions related to the assessment of children’s oral health and oral hygiene habits. Additionally, one question group sought to obtain data on children’s eating habits. One of the question groups was formulated with the aim of acquiring information on the oral health and children’s habits at the earliest age (when parents/guardians started brushing children’s teeth and similar).

#### 2.2.3. Statistical Methods of Data Processing

The collected research data were stored in a database in Microsoft Office Excel 2016 program and processed by a personal computer using the statistical program Statistica 13.1. Descriptive statistics (median, interquartile range, lower quartile, upper quartile, minimum value, maximum value, mean value, and standard deviation) were calculated for all three groups of respondents (SOS Children’s Village, rural area, and urban area). The normality of the distribution of a separate group of data was tested by the Kolmogorov–Smirnov test, which showed that the data did not follow the normal distribution. In accordance with the results obtained by the Kolmogorov–Smirnov test, a nonparametric Kruskal–Wallis test was used to test possible statistically significant difference between the three groups of subjects. For the analysis of data collected by completing the questionnaire, the principal component analysis method (PCA) was used, whereby the value of 0.7 was taken for the lower limit of the factor loading.

## 3. Results

### 3.1. Basic Data on Respondents

A total of 620 children were included in the research, out of which a total of 318 were female and 302 male. A total of 218 children from the SOS Village, 224 from the rural area, and 178 children from the urban area were examined. The average age of the respondents for the examined groups of children from the SOS Children’s Village, children from rural areas, and children from urban areas was 10.3 ± 2.3, 10.2 ± 2.2, and 10.3 ± 2.4 years of age. Children of all examined groups show normal BMI, i.e., have normal body weight (Table 1).

### 3.2. Comparison of Carious, Extracted and Filled Teeth between Subjects with Respect to the Place of Residence

Table 2 shows equal values of median for the component of carious teeth (dt), while significantly, the highest maximum value of this component was recorded in the group of children living in urban areas (*p* = 0.01) compared to the children from the SOS Children’s Village and rural area.

The median components of carious tooth surfaces (ds) of children from rural areas show a slightly higher value compared to children from the SOS Children’s Village and urban areas, while a significantly higher maximum value of the same component was recorded in the group of children from urban areas compared to other groups of children (*p* = 0.01). Significantly lower maximum values of the DT component were recorded in children of the SOS Children’s Village and rural areas compared to children in urban areas (*p* = 0.02). The children of the SOS Children’s Village had a significantly higher maximum value of the MT component compared to other groups of children (*p* = 0.01). For the component of teeth with filling (FT), in children of urban and rural areas significantly higher maximum values were recorded, compared to the children from the SOS Children’s Village (*p* = 0.01). Additionally, for the component of carious surfaces of teeth (DS), significantly higher maximum values were recorded in children of rural and urban areas compared to the children from SOS Children’s Village (*p* = 0.01). The children of the SOS Children’s Village had a significantly higher maximum value of the MS component compared to other groups of children (*p* = 0.01). For the component of tooth surfaces with filling (FS) significantly higher maximum values were recorded in children from urban and rural areas compared to children from the SOS Children’s Village (*p* = 0.01) (Table 2).

### 3.3. Values of dft, dfs, DMFT, DMFS, and SiC Index in Respondents with Regard to the Place of Residence

Table 3 shows the mean values of the dft/dfs index for the children living in the SOS Children’s Village and the children from the rural and urban areas, which indicates that these indices with the SOS Children’s Village respondents show lower mean values (2.42/3.31) compared to the children from other surveyed groups, rural area (3.20/5.58) and urban area (3.67/5.70).

The mean value of the DMFT index of children living in urban areas is higher (2.31) compared to the children in rural areas (1.77) as well as children of the SOS Children’s Village with the lowest mean value of the DMFT index (1.61). The mean values of the DMFS index are approximately the same for all three examined groups of children (Table 3). The results of the Kruskal—Wallis test show a significant difference (*p* = 0.01) in the mean values of the SiC index between the children from the SOS Children’s Village (3.97) and the children from rural areas (4.38) compared to the children from urban areas (5.97) (Table 3).

### 3.4. Analysis of Questionnaire Results by Principal Component Analysis Method

#### 3.4.1. Analysis of the Results of the Questionnaire for Children from the SOS Children’s Village

The following interpretation was obtained by factor analysis of the questionnaire filled in by mothers/guardians for the group of children from the SOS Children’s Village. The multidimensional space of 28 variables was reduced to six latent mutually independent variables of the principal components, i.e., factors (Table 4 and Table 5); six eigenvalues explain a total of 51.09% of the variance. The first factor explains 11.14% of the variance and singles out oral hygiene as the main component. The contribution of this component is the highest, because the eigenvalue of the first factor is 3.12. The second factor explains a further 10.54% of the variance and also singles out the oral hygiene of children and this explains a total of 21.68% of the variance. The eigenvalue of the second factor is 2.95. The third factor singles out the assessment of children’s oral health and explains a further 8.40%, and thus explains a total of 30.08% of the variance with an eigenvalue of 2.35. The fourth factor explains 8.04% of the variance and also highlights the assessment of children’s oral health, and thus explains a total of 38.12% of the variance. The eigenvalue of the fourth factor is 2.25. The fifth factor highlights the eating habits of children and explains a further 6.96%, and thus explains a total of 45.08% of the variance with an intrinsic value of 1.95. The sixth factor explains 6.01% of the variance with the allocation of the socio-economic status of the family, and thus explains a total of 51.09% of the variance with an eigenvalue of 1.68.

#### 3.4.2. Analysis of the Results of the Questionnaire for Children from Rural Areas

The factor analysis of the questionnaire completed by mothers/guardians for a group of children from rural areas resulted in the following interpretation. The multidimensional space consisting of 28 variables was reduced to six latent mutually independent variables of the main components, i.e., factors (Table 6 and Table 7). The six eigenvalues explain a total of 50.97% of the variance. The first factor explains 13.91% of the variance and singles out oral hygiene of children as the main component. The contribution of this component is the highest due to the fact that the eigenvalue of the first factors is 3.89. The second factor explains a further 9.90% of the variance and singles out the assessment of children’s oral health and thus explains a total of 23.81% of the variance. The eigenvalue of the second factor is 2.77. The third factor also singles out the assessment of children’s oral health and thus explains a further 8.23% and the total of 2.04% of the variance with the eigenvalue of 2.30. The fourth factor explains 7.40% of the variance and singles out the socioeconomic status of the family and thus explains the total of 39.44% of the variance. The eigenvalue of the fourth factor is 2.07. The fifth factor singles out children’s eating habits and thus explains a further 5.87% and the total of 45.31% of the variance with an eigenvalue of 1.64. The sixth factor explains 5.66% of the variance with also singling out children’s eating habits and thus explaining the total of 50.97% of the variance with an eigenvalue of 1.58.

#### 3.4.3. Analysis of the Results of the Questionnaire for Children from Urban Areas

The following interpretation was obtained by the factor analysis of the questionnaire completed by mothers/guardians for the group of children from the urban area. The multidimensional space consisting of 28 variables was reduced to six latent mutually independent variables of the main components, i.e., factors (Table 8 and Table 9). Six eigenvalues explain the total of 53.05% of the variance. The first factor explains 15.37% of the variance and singles out the socioeconomic status of the family as the main component. The contribution of this component is the highest, because the eigenvalue of the first factor is 4.30. The second factor explains a further 9.64% of the variance and singles out children’s oral hygiene and thus explains the total of 25.01% of the variance. The eigenvalue of the second factor is 2.70. The third factor singles out the assessment of children’s oral health and explains a further 8.64% and thus explains the total of 33.65% of the variance with an eigenvalue of 2.42. The fourth factor explains 7.45% of the variance and singles out the assessment of children’s oral health and thus explains the total of 41.10% of the variance. The eigenvalue of the fourth factor is 2.09. The fifth factor singles out children’s eating habits and explains a further 6.22% thus explaining the total of 47.32% of the variance with an eigenvalue of 1.74. The sixth factor explains 5.73% of the variance also singling out children’s eating habits and thus explains the total of 53.05% of the variance with an eigenvalue of 1.61.

#### 3.4.4. Comparative Analysis of the Exclusion Factors of the Respondents

By the research of oral health of primary school children of the SOS Children’s Village compared to the children of rural and urban areas, who live with their biological mothers, and the application of the analysis of main components with rotation of factors, it was attempted to reduce a number of variables to a smaller number of basic variables describing oral health. After the analysis of the main components, the interpretation of the factors starts with the matrix of the factor structure after the orthogonal varimax factor rotation and the identification of variables that have high absolute factor loadings on the same factor. After the factor analysis of the questionnaires completed by mothers from the SOS Children’s Village and biological mothers from rural areas as well as biological mothers from urban areas, it can be concluded that for each of the examined groups, there are six exclusion factors and the belonging variables with their factor loadings that describe the condition of the oral health for each of the exclusion factors (Table 10).

## 4. Discussion

It is a known fact that oral health problems in primary school children and early adolescent age have an impact on their psychophysical growth and development. Children with oral problems feel less valuable, have lower self-esteem, and are more shy in social contact, sad, and depressive, compared to the children without oral problems [16]. Untreated caries at primary school age lead to premature tooth loss, which can lead to developmental disorders, developing difficulties of speaking skills, and reduced self-confidence in children. Children are often unable to verbalize dental pain. It can frequently be noticed that children have learning difficulties, i.e., that they show signs of pain such as anxiety, fatigue, irritability, depression, and avoidance of daily obligations. This condition of a child is often not recognized by their environment [17].

A study by Seirawan at al. indicated that the children living in lower-income families, who needed dental care in the last 12 months were three times more absent from school than other children [18]. The family is the most important environment that provides a child with physical, mental, and social growth, as well as knowledge, skills, and attitudes. Considering the importance of the foundations for these competences for further growth, their quality formation in early childhood produces best results. The research conducted with the sample of 504 children in Tehran indicated the importance of mother’s role in the oral health of a child, where mothers, by their example, give importance to and adopt good oral hygiene habits, leading to more frequent similar habits in children [19].

Comparing the quality of life of children living with parents and children with no parental care in relation to oral health and caries status, the results have demonstrated that the children without parental care have a lower quality of life compared to children who live with parents [20].

The results of the research show that well-developed oral hygiene habits of children living with SOS mothers in the SOS Children’s Village in Croatia have an impact on the lower DMFT index values (1.61) compared to the children living with biological mothers in the rural (1.77) and urban (2.31) areas. The biggest problems of children’s oral health in Croatia are dental fear and high caries incidence. The fact that Croats consume 3.5 toothpastes and 0.6 per year indicates that, in Croatia, there is no sufficiently developed awareness on the importance of oral health preservation [21].

The research conducted in Croatia in 2003 shows that only 10.7% of children aged 11 to 14 brush their teeth more than once per day, 44.05% occasionally, 44.93% rarely, and 0.44% never. DMFT index for the target population in the mentioned research was 6.7 [22]. In Croatia, in 1991, DMFT index was 2.6, and in 1999 3.5. The research from 2007 indicates that the value of the same index was 9.5 [23]. The study by Dukić et al. shows that the mean DMFT/DMFS index values recorded in the urban area (the city of Zagreb), in the period from 2009 to 2020, for the group of children aged 7 to 15, were 4.1/5.6., while in our research, the children from urban areas had lower DMFT/DMFS index values (2.31/2.44). SiC index value in the research by Dukić et al. was also higher (7.4) compared to our research (5.97) [24]. In a study by Jurić et al. from 2008, dft/DMFT (7.7/6.7) and dfs/DMFS (16.5/11.8) results for a group of children from rural and sub-rural areas were higher compared to the rural area included in our research, where lower dft/DMFT (3.20/1.77) values were recorded, i.e., dfs/DMFS (5.58/2.40). Additionally, SiC index value (4.38) in our research is lower compared to the previously mentioned research, where it was 10.89 [25]. According to the data of the Central Health Information System of the Republic of Croatia from 2013 to 2015, the DMFT index in twelve-year-olds was 4.18 [26,27]. In a study by Lešić et al. conducted on a population of children from rural and urban areas in Croatia, DMFT index was 2.4 in rural areas and 2.0 in urban areas [28]. The most recent research on the prevalence of caries in Croatia shows DMFT values of 1.2 [29].

The research conducted in the neighboring Bosnia and Herzegovina in 2008 by Muratbegović et al. showed that the mean value of the DMFT index in 12-year-olds was 4.16, and the SiC index value was 7.41 [30]. A higher mean value of DMFT index was measured in Monte Negro in twelve-year-olds (3.43) as well as a higher SiC index value (6.0) compared to our research [31]. Other neighboring countries have also shown higher DMFT index values in twelve-year-olds: Serbia 7.8, Macedonia 3.5, and Albania 2.6 [1,32].

The obtained DMFT/DMFS index values in children from the SOS Children’s Village in Croatia are lower than the average in the research in previously mentioned countries, which is probably a result of better awareness on maintaining oral hygiene as well as better oral health assessment by SOS mothers for the children they care about. In contrast to European countries with a high DMFT index, a study by Gatou et al., conducted among Greek twelve-year-olds, showed a DMFT index of 1.35 [33]. A research conducted among Portugese twelve-year-olds showed a DMFT index of 1.18 and a SiC index of 2.68 [34], while in Germany the lowest values of DMFT (0.71) and SiC index (2.29) were recorded [35]. The data on the incidence of caries in populations of children of the SOS Children’s Villages in the world are lacking. The only known results of the research relate to the SOS Children’s Village Bhopal in India, where children under the age of 20 were included in the research. The results of this study show that the value of the DMFT within the group of children aged 11 to 15 was 2.92 [36], while in our study a significantly lower DMFT index of 1.61 was recorded. Since carious lesions in the group of school age children occur on the occlusal surfaces of the first permanent molars, and later on the approximal surface of the posterior teeth, special attention is required in the process of diagnosing. Additionally, the research conducted among the children grown up in foster families indicates that caries most frequently occurs on lower permanent and deciduous teeth [37]. The focus of numerous previous studies were the effects of different factors on the oral health of children [2,38,39,40].

It is evident in Table 10 that in the groups of children from the SOS Children’s Village and from the rural area compared to the children from the urban area, oral hygiene was singled out as the most important factor, which is in accordance with the recorded values of the DMFT index in the examined groups of children. As an equally important factor for all three groups of respondents, the assessment of oral health as well as eating habits have been singled out, so it can be claimed that they have equal influences on the oral statues of the examined groups of children. In the group of children from the SOS Children’s Village, the least significant factor is the socioeconomic status, which is somewhat more significant for the children from the rural area, and the most significant for the children from the urban area. Given the fact that this is a specific life of mothers and children in the SOS Children’s Village (approximately equal monthly income of SOS mothers per SOS house), the factor of the socioeconomic status does not have as great an influence as it does on the lives of children living in the families with different socioeconomic statuses.

## 5. Conclusions

The population of primary school children living in the SOS Children’s Village has lower dft/DMFT and dfs/DMFS index values compared to the children from biological families from rural and urban areas. The SiC index for the population of primary school children living in the SOS Children’s Village indicates a lower value compared to the children from biological families from the rural and urban areas. The population of children from the rural area has lower dft/DMFT index values compared to the children from the urban area. The oral health assessment by the SOS mothers does not differ compared to the assessment by biological mothers of children from the rural and urban areas.

## Figures and Tables

**Table 1:** Basic data on respondents.

Variable	SOS Children’s Village	Rural Area	Urban Area
Total respondents, N	218	224	178
Total female, N (%)	105 (48.16)	119 (53.13)	94 (52.81)
Total male, N (%)	113 (51.84)	105 (46.87)	84 (47.19)
Average age of respondents (years), MV ± SD	10.3 ± 2.3	10.2 ± 2.2	10.3 ± 2.4
Average weight (kg), MV ± SD	39.98 ± 12.63	43.04 ± 15.38	41.63 ± 13.67
Average height (m), MV ± SD	1.48 ± 0.15	1.49 ± 0.13	1.48 ± 0.16
BMI, MV ± SD	17.77 ± 2.85	18.88 ± 4.11	18.57 ± 3.33

BMI—body mass index; MV—mean value; SD—standard deviation.

**Table 2:** Comparison of carious (dt/ds/DT/DS), extracted (MT/MS) and filled teeth (ft/fs/FT/FS) between subjects with respect to the place of residence.

Component	SOS Children’s Village				Rural Area				Urban Area				Kruskal-Wallis, H	*p*
	M	IQR	Min	Max	M	IQR	Min	Max	M	IQR	Min	Max		
dt	1	4	0	9	2	3	0	11	1	5	0	14	12.75	0.01
ft	0	1	0	4	0	1	0	5	0	1	0	4	2.36	0.31
ds	1	5	0	12	3	6	0	25	1	6	0	38	16.87	0.01
fs	0	1	0	6	0	1	0	6	0	1	0	5	2.59	0.27
DT	0	1	0	8	0	2	0	8	0	1	0	13	7.96	0.02
MT	0	0	0	2	0	0	0	0	0	0	0	0	11.15	0.01
FT	0	1	0	6	0	0	0	9	0	2	0	11	19.86	0.01
DS	0	1	0	10	0	2.5	0	17	0	1	0	13	8.82	0.01
MS	0	0	0	2	0	0	0	0	0	0	0	0	11.15	**0.01**
FS	0	1	0	8	0	0	0	10	0	2	0	11	15.67	**0.01**

M—median; IQR—interquartile range; Min—minimum value; Max—maximum value; dt—carious deciduous tooth; ft—deciduous tooth with filling; ds—carious surface of deciduous tooth; fs—surface of the deciduous tooth with filling; DT—carious permanent tooth; MT—extracted permanent tooth; FT—permanent tooth with filling; DS—carious surface of permanent tooth; MS—surfaces of extracted teeth; FS—surface of a permanent tooth with filling.

**Table 3:** dft, dfs, DMFT, DMFS, and SiC index values.

Index	SOS Children’s Village	Rural Area	Urban Area
dft	2.42	3.20	3.67
dfs	3.31	5.58	5.70
DMFT	1.61	1.77	2.31
DMFS	2.23	2.40	2.44
SiC	3.97	4.38	5.97

dft—decayed filled tooth; dfs—decayed filled surfaces; DMFT—decayed missing filled tooth; DMFS—decayed missing filled surfaces; SiC—significant caries index.

**Table 4:** Eigenvalues, percentages of explained variance, and cumulative percentages of variance for the group of children from the SOS Children’s Village.

Factor	Eigenvalue	Percentage of Explained Variance	Cumulative Percentages of Variance
1.	3.12	11.14	11.14
2.	2.95	10.54	21.68
3.	2.35	8.40	30.08
4.	2.25	8.04	38.12
5.	1.95	6.96	45.08
6.	1.68	6.01	51.09

**Table 5:** Matrix of the rotated factor structure for a group of children from the SOS Children’s Village.

Variable	Factors					
	1	2	3	4	5	6
1. How would you describe the health of your child’s teeth or mouth?	0.0032	0.1345	−0.0538	**−0.7185**	0.1168	−0.0755
2. How much is your child’s overall well-being affected by the condition of his/her teeth, lips, jaws, or mouth?	0.2604	0.0771	−0.0109	−0.1509	0.3494	−0.0192
3. At this stage of growing up, are your child’s teeth crooked at all?	0.1507	0.1256	−0.2458	0.5142	0.1127	0.0505
4. At the moment, do you think your child’s teeth are alright as they are, or would you prefer him/her to have them straightened?	−0.2592	−0.1264	0.0137	**−0.7715**	−0.0871	−0.0684
5. In general, compared to other children, what do your child’s teeth look like?	0.0227	0.0904	−0.1005	**−0.7916**	−0.1202	0.1641
6. In the last 12 months, has your child had any fillings placed in his/her teeth?	−0.0539	−0.1833	0.0866	0.0896	0.0763	0.0445
7. In the last 12 months, have any of your child’s teeth been removed because of tooth decay or had a toothache?	−0.0791	−0.0299	**0.7167**	0.2204	0.0106	−0.5061
8. During the last 12 months, how often has child had a toothache?	0.0164	0.0635	**0.7900**	0.0444	0.1273	0.0224
9. How often has child had to avoid eating some foods because of problems with his/her teeth or mouth during the last 12 months?	0.0327	0.0759	**0.8352**	−0.0064	−0.0794	−0.0728
10. How old was child when he/she first started having his/her teeth brushed?	**0.8476**	−0.0678	0.0484	0.0918	−0.0010	0.0275
11. How old was child when he/she started cleaning his/her teeth on his/her own?	**0.8794**	0.0107	0.0233	−0.0404	0.0789	0.0528
12. How old was child when he/she started brushing with toothpaste?	**0.8628**	0.0257	−0.0311	0.1328	0.0047	−0.0575
13. How often are child’s teeth brushed?	0.0175	−0.0077	−0.1171	0.0908	0.5990	0.2137
14. What type of toothpaste does your child use?	0.0805	**−0.8666**	−0.0064	0.0001	−0.1025	−0.0108
15. What size of toothbrush does your child use?	0.0103	**0.8757**	0.1105	0.0156	0.0017	−0.0968
16. Does child, or did child when an infant, go to bed or for a nap with a drink?	−0.0334	−0.2878	0.2128	−0.0391	0.0369	0.1020
17. How often does child eat something between his/her main meals?	0.0855	−0.0024	0.4122	0.0650	−0.0227	0.5209
18. In the last week, how often would child take something to drink in bed or during the night?	−0.0430	0.0337	−0.0666	-0.1099	**−0.7200**	0.2563
19. In the last week, what did child take to drink in bed or during the night?	0.1094	0.1795	0.1825	0.1292	**−0.7771**	0.2756
20. In the last week, how often did child take something to eat in bed or during the night?	−0.1565	−0.3099	0.2153	−0.2792	0.0447	0.0264
21. Thinking about the first time that child visited a dental professional, how old was he/she then?	0.0751	0.5141	−0.0622	−0.4031	−0.0571	−0.0316
22. Why did child go to this dental professional for the first time?	−0.0031	−0.4195	−0.0040	0.0195	0.2642	−0.1737
23. How long has it been since child last visited a dental professional for any reason?	0.0591	−0.1397	0.4138	−0.1393	−0.1505	0.3067
24. What was the reason for child’s last dental visit?	0.1877	0.4365	−0.0794	−0.1949	−0.1135	0.0082
25. What is your school qualification?	−0.0209	−0.0821	−0.0734	0.0115	0.0424	**0.8558**
26. Which of these statements best describes your employment status now?	0.0145	0.1561	0.1171	−0.0362	0.3144	−0.0733
27. How many people are in this household?	0.0359	0.0306	−0.0367	0.0867	0.0112	**−0.8432**
28. What is the total income that your household got from all sources in the last 12 months?	0.1470	0.3202	0.1185	0.0410	0.5609	0.1499

Bold—statistically significant value of factor loading. Factor loading—indicates the importance of each of the variables for particular isolated factors.

**Table 6:** Eigenvalues, percentages of the explained variance, and cumulative percentages for the group of children from rural area.

Factor	Eigenvalue	Percentage Explained Variance	Cumulative Percentages of Variance
1.	3.89	13.91	13.91
2.	2.77	9.90	23.81
3.	2.30	8.23	32.04
4.	2.07	7.40	39.44
5.	1.64	5.87	45.31
6.	1.58	5.66	50.97

**Table 7:** Rotated matrix of factor structure for the group of children from rural areas.

Variable	Factors					
	1	2	3	4	5	6
1. How would you describe the health of your child’s teeth or mouth?	0.1907	−0.5529	−0.1600	−0.1932	−0.1019	0.0869
2. How much is your child’s overall well-being affected by the condition of his/her teeth, lips, jaws, or mouth?	0.1996	−0.1665	**0.7197**	0.2377	0.0105	0.1586
3. At this stage of growing up, are your child’s teeth crooked at all?	0.3178	−0.0809	0.4787	−0.1506	0.0564	0.0038
4. At the moment, do you think your child’s teeth are alright as they are or would you prefer him/her to have them straightened?	−0.1019	−0.1221	**−0.7017**	0.0433	−0.0665	0.0071
5. In general, compared to other children, what do your child’s teeth look like?	0.2344	0.0147	**−0.7707**	0.0408	−0.0614	0.0445
6. In the last 12 months, has your child had any fillings placed in his/her teeth?	0.1198	0.4074	−0.0149	−0.1032	−0.0342	0.0927
7. In the last 12 months have any of your child’s teeth been removed because of tooth decay or had a toothache?	−0.0211	**0.7376**	0.3149	0.0355	−0.0012	0.1211
8. During the last 12 months how often has child had a toothache?	0.0422	**0.8313**	−0.0866	0.0069	0.0901	−0.0679
9. How often has child had to avoid eating some foods because of problems with his/her teeth or mouth during the last 12 months?	−0.0851	**0.8050**	0.0039	0.0215	−0.0475	−0.0056
10. How old was child when he/she first started having his/her teeth brushed?	**0.7682**	−0.1185	−0.0441	−0.1258	−0.2253	−0.0375
11. How old was child when he/she started cleaning his/her teeth on his/her own?	**0.8299**	0.0698	−0.0769	0.0606	0.0386	−0.0415
12. How old was child when he/she started brushing with toothpaste?	**0.8538**	−0.0403	0.0920	−0.1290	−0.0395	−0.0037
13. How often are child’s teeth brushed?	−0.2477	0.1143	0.0352	0.0848	0.1877	0.1312
14. What type of toothpaste does your child use?	−0.0108	−0.0845	−0.0163	−0.2074	0.0471	0.0880
15. What size of toothbrush does your child use?	−0.0112	0.1495	0.0853	−0.0514	−0.1586	0.1932
16. Does child, or did child when an infant, go to bed or for a nap with a drink?	−0.2151	0.0129	0.0682	0.1124	**0.7798**	0.1289
17. How often does child eat something between his/her main meals?	0.2027	0.2433	0.2347	0.0361	0.1113	**0.7004**
18. In the last week, how often would child take something to drink in bed or during the night?	−0.1951	0.2207	0.1101	0.0773	**0.7029**	0.2502
19. In the last week, what did child take to drink in bed or during the night?	0.1155	−0.0224	0.0128	−0.0704	**0.7378**	0.0730
20. In the last week, how often did child take something to eat in bed or during the night?	−0.1471	−0.1035	−0.0647	0.1718	0.1449	**0.7759**
21. Thinking about the first time that child visited a dental professional, how old was he/she then?	0.4302	−0.0335	−0.0861	0.1542	−0.4619	−0.0259
22. Why did child go to this dental professional for the first time?	−0.1532	0.0732	0.0693	0.2146	0.5469	−0.4584
23. How long has it been since child last visited a dental professional for any reason?	0.2708	0.1255	0.0019	0.0236	−0.0384	−0.0384
24. What was the reason for child’s last dental visit?	0.1472	−0.1998	0.1584	−0.1972	−0.1083	0.0708
25. What is your school qualification?	−0.1002	−0.0148	−0.1189	**0.8298**	0.0685	−0.1331
26. Which of these statements best describes your employment status now?	0.1494	0.0644	−0.1022	**−0.7108**	0.0402	−0.4334
27. How many people are in this household?	0.3715	−0.2341	−0.0741	−0.1431	0.2376	−0.1204
28. What is the total income that your household got from all sources in the last 12 months?	0.0078	0.1276	0.0412	**0.8042**	0.0079	0.2039

Bold—statistically significant value of factor loading. Factor loading—indicates the importance of each variable for particular isolated factors.

**Table 8:** Eigenvalues, percentages of explained variance, and cumulative percentages for the group of children from the urban area.

Factor	Eigenvalue	Percentage of Explained Variance	Cumulative Percentages of Variance
1.	4.30	15.37	15.37
2.	2.70	9.64	25.01
3.	2.42	8.64	33.65
4.	2.09	7.45	41.10
5.	1.74	6.22	47.32
6.	1.61	5.73	53.05

**Table 9:** Rotated matrix of factor structure for the group of children from urban area.

Variable	Factors					
	1	2	3	4	5	6
1. How would you describe the health of your child’s teeth or mouth?	−0.2061	0.2156	**0.7289**	−0.2777	−0.1814	0.0126
2. How much is your child’s overall well-being affected by the condition of his/her teeth, lips, jaws, or mouth?	0.0947	0.1102	0.0673	0.0073	0.1878	0.5858
3. At this stage of growing up, are your child’s teeth crooked at all?	−0.2458	0.0746	**−0.7490**	−0.3624	0.0878	0.0497
4. At the moment, do you think your child’s teeth are alright as they are or would you prefer him/her to have them straightened?	−0.0149	−0.0226	**0.8788**	−0.0051	0.0289	−0.0426
5. In general, compared to other children, what do your child’s teeth look like?	−0.0158	0.2313	**0.7161**	−0.0264	−0.0729	0.1432
6. In the last 12 months, has your child had any fillings placed in his/her teeth?	0.0332	−0.0169	0.0514	**0.7413**	0.2515	0.1564
7. In the last 12 months have any of your child’s teeth been removed because of tooth decay or had a toothache?	−0.0813	0.1016	−0.4932	**0.7649**	−0.0331	−0.0201
8. During the last 12 months how often has child had a toothache?	0.1603	−0.1563	−0.1186	0.2184	−0.0541	0.1283
9. How often has child had to avoid eating some foods because of problems with his/her teeth or mouth during the last 12 months?	0.3013	−0.1624	−0.0833	0.2295	−0.3866	0.0811
10. How old was child when he/she first started having his/her teeth brushed?	−0.0456	**0.7794**	0.1041	0.0687	−0.0032	0.3025
11. How old was child when he/she started cleaning his/her teeth on his/her own?	−0.0202	**0.8454**	0.0209	−0.0882	−0.0429	−0.1758
12. How old was child when he/she started brushing with toothpaste?	0.0370	**0.8348**	0.1003	−0.0644	0.0154	−0.0243
13. How often are child’s teeth brushed?	0.2568	−0.1963	−0.0450	0.1076	0.3505	0.0488
14. What type of toothpaste does your child use?	−0.0372	−0.0596	0.1905	0.1258	−0.1597	−0.0309
15. What size of toothbrush does your child use?	−0.0555	0.0411	−0.0062	−0.0245	0.0774	0.0056
16. Does child, or did child when an infant, go to bed or for a nap with a drink?	0.0108	−0.1506	−0.0426	0.1949	**0.7678**	−0.0166
17. How often does child eat something between his/her main meals?	−0.0164	−0.0132	0.0384	0.0379	0.0170	**0.7647**
18. In the last week, how often would child’s take something to drink in bed or during the night?	0.0548	−0.0471	−0.1581	−0.0447	**0.7528**	0.0017
19. In the last week, what did child take to drink in bed or during the night?	0.0739	−0.0069	0.0706	0.0421	**0.7957**	0.0772
20. In the last week, how often did child take something to eat in bed or during the night?	0.0432	0.1140	−0.0403	−0.0708	0.3893	0.0534
21. Thinking about the first time that child visited a dental professional, how old was he/she then?	−0.0661	**0.7176**	−0.1539	0.0675	−0.1352	−0.0761
22. Why did child go to this dental professional for the first time?	0.0772	−0.0059	−0.0801	0.0012	0.1163	0.1122
23. How long has it been since child’s last visited a dental professional for any reason?	−0.1419	0.3442	0.0100	0.0296	−0.1319	0.0111
24. What was the reason for child’s last dental visit?	−0.0899	0.1205	0.0014	**−0.7995**	0.1678	0.0999
25. What is your school qualification?	**0.8700**	0.0866	0.0218	0.0353	0.0845	−0.0274
26. Which of these statements best describes your employment status now?	**−0.7910**	0.2034	−0.1394	0.0226	0.0385	−0.0692
27. How many people are in this household?	0.1435	−0.2610	−0.1195	0.0923	−0.0779	0.5346
28. What is the total income that your household got from all sources in the last 12 months?	**0.8349**	−0.0719	−0.0664	0.0188	0.0307	0.0666

Bold—statistically significant value of factor loading. Factor loading—indicates the importance of each variable for particular isolated factors.

**Table 10:** Comparative presentation of the exclusion factors of the respondents from the SOS Children’s Village, rural, and urban areas.

Factor	Factor Label		
	SOS Children’s Village	Rural Area	Urban Area
1.	Oral hygiene	Oral hygiene	Socioeconomic status
2.		Oral health assessment	Oral hygiene
3.	Oral health assessment		Oral health assessment
4.		Socioeconomic status	
5.	Eating habits	Eating habits	Eating habits
6.	Socioeconomic status		

## Data Availability

The data presented in this study are available on request from the corresponding author.
